# Preoperative BUN-to-Albumin Ratio Is Independently Associated with Major Reamputation After Distal Amputation in Diabetic Foot: A Retrospective Cohort Study

**DOI:** 10.3390/jcm15093279

**Published:** 2026-04-25

**Authors:** Bahri Bozgeyik, Erman Öğümsöğütlü, Murat Düzgün, Gazi Huri

**Affiliations:** 1 Department of Orthopaedics and Traumatology, Faculty of Medicine, Gaziantep University, 27310 Gaziantep, Turkey; muratduzgun1995@gmail.com; 2 Department of Orthopaedics and Traumatology, Yalova Training and Research Hospital, 77200 Yalova, Turkey; eogumsogutlu@gmail.com; 3 Aspetar Orthopaedic and Sports Medicine Hospital, Doha 29222, Qatar; gazihuri@yahoo.com; 4 Department of Orthopaedics and Traumatology, Hacettepe University, 06230 Ankara, Turkey

**Keywords:** diabetic foot, reamputation, CRP, albumin, BUN

## Abstract

**Background:** Major level escalation following distal amputation for diabetic foot—defined as subsequent below-knee amputation (BKA)—represents a clinically meaningful endpoint with substantial functional implications. Identifying reliable and readily available preoperative biomarkers capable of predicting major level escalation remains a clinical challenge. This study aimed to evaluate the independent prognostic value of the C-reactive protein-to-albumin ratio (CAR) and blood urea nitrogen-to-albumin ratio (BAR) in predicting postoperative major level escalation. **Methods:** We retrospectively analyzed 151 consecutive patients who underwent distal lower extremity amputation for diabetic foot between January 2020 and October 2025. The primary outcome was ipsilateral below-knee amputation within the follow-up period. Preoperative CAR and BAR were calculated using laboratory parameters obtained within 24 h prior to surgery. Given the shared albumin component, two separate multivariable logistic regression models were constructed to evaluate independent associations, adjusting for age, peripheral arterial disease (PAD), and index amputation level. **Results:** During follow-up, 46 patients (30.5%) required major level escalation (BKA). Both CAR and BAR were significantly higher in patients who developed BKA (*p* < 0.001 and *p* = 0.006, respectively). In receiver operating characteristic (ROC) analysis, CAR demonstrated acceptable discriminative ability (AUC = 0.745; 95% CI 0.653–0.827), whereas BAR showed modest performance (AUC = 0.640; 95% CI 0.536–0.738). The optimal cutoff values were 1.50 for CAR (sensitivity 76.1%, specificity 61.9%) and 0.61 for BAR (sensitivity 73.9%, specificity 44.8%), although these thresholds were considered exploratory. In the primary multivariable analysis, both CAR (OR 1.16; 95% CI 1.02–1.32; *p* = 0.024) and BAR (OR 4.02; 95% CI 1.85–8.73; *p* = 0.005) were independently associated with major level escalation. In sensitivity analyses, BAR retained independent significance, whereas CAR did not (*p* = 0.100). **Conclusions:** Preoperative BAR demonstrated robust independent association with major level escalation across both primary and sensitivity analyses, while CAR showed association in the primary model only. These composite biomarkers may provide hypothesis-generating insights into systemic risk profiling in diabetic foot surgery, pending external validation.

## 1. Introduction

Diabetic foot disease represents one of the most devastating and clinically complex complications of diabetes mellitus, frequently resulting in infection, impaired wound healing, and lower extremity amputation [1]. With the global prevalence of diabetes steadily increasing, amputation rates related to diabetic foot infections continue to pose a major public health burden [2]. Beyond their association with increased mortality and morbidity, lower extremity amputations substantially reduce functional independence and impose significant socioeconomic costs on healthcare systems.

Distal amputations are primarily performed with the intention of preserving limb length and maximizing functional potential. However, they do not always provide definitive resolution. Failure of wound healing, persistent infection, or underlying vascular insufficiency may necessitate more proximal reamputation, commonly referred to as level escalation [3]. Escalation of amputation level represents a clinically critical endpoint, as it directly and permanently affects patient mobility, prosthetic adaptation, and overall quality of life. Despite advances in multidisciplinary diabetic foot management, determining the optimal amputation level remains one of the most challenging aspects of surgical decision-making. Current clinical practice relies on vascular assessments, infection severity, imaging findings, and surgical judgment; however, these parameters do not consistently and reliably predict postoperative major level escalation or the subsequent need for more proximal amputation [4]. Consequently, there is an increasing need for objective, reproducible, and easily accessible perioperative prognostic biomarkers.

Systemic inflammation and nutritional status play central roles in wound healing and infection control in diabetic foot disease [5]. Serum albumin has long been recognized as a marker of nutritional status and chronic inflammatory burden, and hypoalbuminemia has been associated with impaired wound healing and adverse surgical outcomes [6]. Nevertheless, albumin alone may not adequately reflect the acute inflammatory response or metabolic stress accompanying severe infection.

The C-reactive protein-to-albumin ratio (CAR) has emerged as a composite biomarker integrating systemic inflammatory activity and nutritional status. Previous studies have suggested that CAR may be associated with amputation risk in patients with diabetic foot infections [7]. However, most available investigations have focused on the presence or absence of amputation rather than the need for more proximal reamputation following an initial distal procedure—an outcome with more direct implications for long-term functional prognosis. Blood urea nitrogen (BUN), on the other hand, reflects catabolic stress, renal perfusion alterations, and systemic metabolic load [8]. When evaluated in conjunction with albumin, it may provide a more comprehensive assessment of physiological reserve and tissue healing capacity [9]. Although the BUN-to-albumin ratio (BAR) has demonstrated prognostic value in various inflammatory and critical illness settings, its role in predicting postoperative level escalation after distal amputation for diabetic foot has not been clearly defined in the literature [10].

Accordingly, the present study aimed to evaluate the prognostic significance of CAR and BAR in predicting major level escalation following distal amputation in patients with diabetic foot. We hypothesized that these inflammation- and nutrition-based composite biomarkers would be independently associated with major level escalation following distal amputation, even after adjustment for relevant demographic and clinical factors.

## 2. Materials and Methods

This retrospective observational cohort study was conducted at Gaziantep University Faculty of Medicine, Şahinbey Research and Application Hospital, to evaluate the prognostic value of the blood urea nitrogen-to-albumin ratio (BAR) and the C-reactive protein-to-albumin ratio (CAR) in predicting postoperative level escalation following distal lower extremity amputation for diabetic foot. The study protocol was approved by the Gaziantep University Non-Interventional Clinical Research Ethics Committee (Approval No: 2026/03) and was carried out in accordance with the principles of the Declaration of Helsinki. Given the retrospective design and the use of anonymized data, the requirement for written informed consent was waived.

Patients who underwent distal amputation at the level of the foot or ankle for diabetic foot between January 2020 and October 2025 were retrospectively identified through the institutional hospital information system. To prevent duplication and repeated measures, only the index (first) amputation during the study period was included for each patient. Inclusion criteria were age ≥18 years, distal amputation (toe, ray, Lisfranc, Chopart, or Syme level) performed for diabetic foot infection, availability of complete preoperative laboratory data, and a minimum postoperative follow-up of six months. Patients with traumatic amputations, active malignancy, chronic systemic inflammatory disease, end-stage renal failure, dialysis dependency, or incomplete clinical or laboratory data were excluded. These exclusion criteria were applied to minimize the influence of independent systemic conditions that could affect inflammatory markers and serum albumin levels. Follow-up duration was defined as the time from the index amputation to reamputation or the last outpatient evaluation. To minimize the potential impact of heterogeneous follow-up durations inherent to the retrospective study design, outcome assessment was anchored to a six-month time horizon. All major reamputation events in the cohort occurred within the first six months following the index amputation. Patients with incomplete clinical or laboratory data required for analysis were excluded from the study. Missing data were primarily related to incomplete laboratory measurements, and no imputation methods were applied due to the retrospective design.

Demographic characteristics (age and sex) and relevant clinical comorbidities, including hypertension, smoking status, and peripheral arterial disease (PAD), were recorded from patient files. The diagnosis of PAD was confirmed based on documented clinical examination findings and, when available, Doppler ultrasonography and/or computed tomography angiography reports. Decisions regarding amputation and determination of amputation level were made by a multidisciplinary team comprising specialists in orthopedics and traumatology, endocrinology, infectious diseases, plastic surgery, and cardiovascular surgery. Ulcer severity was assessed according to the Wagner classification system. Patients with Wagner grade 2 or higher neuropathic or combined ulcers were considered candidates for amputation, whereas Wagner grade 0–1 ulcers and isolated vasculopathic ulcers were excluded to ensure a more homogeneous study population.

All laboratory parameters were obtained at hospital admission or within 24 h prior to surgery as part of routine biochemical and hematological analyses. Recorded variables included HbA1c (%) to assess glycemic control; complete blood count components, including white blood cell count (WBC, /µL), neutrophil count (/µL), lymphocyte count (/µL), and platelet count (/µL); renal function and catabolic load indicators, including blood urea nitrogen (BUN, mg/dL) and creatinine (mg/dL); inflammatory markers, including C-reactive protein (CRP, mg/L) and erythrocyte sedimentation rate (ESR, mm/h); and serum albumin (g/L).

The BUN-to-albumin ratio (BAR) was calculated by dividing BUN (mg/dL) by serum albumin (g/L), and the CRP-to-albumin ratio (CAR) was calculated by dividing CRP (mg/L) by serum albumin (g/L). These ratios were considered composite biomarkers reflecting systemic inflammatory burden as well as nutritional and metabolic reserve. Although the primary analytical focus of the study was the prognostic significance of CAR and BAR, other laboratory parameters were analyzed as supportive clinical variables in group comparisons.

The primary outcome was defined as ipsilateral below-knee amputation (BKA) following the index distal amputation. In this cohort, all cases of level escalation resulted in below-knee amputation, thereby representing a clinically meaningful major endpoint. Follow-up duration was calculated from the date of the index amputation to the date of reamputation or the last outpatient evaluation. All included patients had a minimum follow-up of six months. Patients were categorized into two groups according to whether they required major level escalation during the follow-up period.

### Statistical Analysis

All statistical analyses were performed using IBM SPSS Statistics for Windows, Version 22.0 (IBM Corp., Armonk, NY, USA). The distribution of continuous variables was assessed using the Shapiro–Wilk test. Normally distributed variables were presented as mean ± standard deviation, whereas non-normally distributed variables were expressed as median (interquartile range). Continuous variables were presented as mean ± standard deviation or median (interquartile range), as appropriate for descriptive purposes. Categorical variables were summarized as counts and percentages. Between-group comparisons were conducted using the independent samples Student’s t-test for normally distributed continuous variables and the Mann–Whitney U test for non-normally distributed variables. Categorical variables were compared using the chi-square test or Fisher’s exact test, as appropriate. The discriminative performance of the BUN-to-albumin ratio (BAR) and the CRP-to-albumin ratio (CAR) for predicting postoperative level escalation was evaluated using receiver operating characteristic (ROC) curve analysis. The area under the curve (AUC) was calculated with corresponding 95% confidence intervals (CI). ROC-derived cutoff values were explored using the Youden index; however, these thresholds were considered exploratory and were not emphasized in the interpretation of results. To identify independent predictors of postoperative level escalation, multivariable logistic regression analysis was performed. Because both CAR and BAR include serum albumin as a shared component, two separate multivariable models were constructed to minimize potential multicollinearity. For regression analyses, index amputation levels were collapsed into clinically and statistically meaningful categories (toe, intermetatarsal, and midfoot/hindfoot). This approach was adopted due to the low number of observations in certain subgroups (e.g., Lisfranc, Chopart, and Syme), in order to reduce sparse-data bias and improve model stability. This categorization was determined prior to multivariable modeling. Although some laboratory variables demonstrated non-normal distributions, CAR and BAR were analyzed in their original (non-transformed) scale to preserve clinical interpretability. In Model A, CAR was evaluated alongside clinical covariates, whereas in Model B, BAR was assessed with the same set of covariates. Model calibration was examined using the Hosmer–Lemeshow goodness-of-fit test. Results were reported as odds ratios (OR) with 95% confidence intervals. Given that the majority of level escalation events occurred within the first six months and that all patients were followed for at least six months, a binary outcome approach was considered methodologically appropriate. Multicollinearity was further assessed using the variance inflation factor (VIF), and all predictors demonstrated VIF values below 2, indicating no significant multicollinearity. Although HbA1c demonstrated a significant difference in univariable analysis, it was not included in the primary multivariable models in order to preserve model parsimony and to minimize the risk of overfitting, in accordance with events-per-variable considerations given the limited number of outcome events. In addition, sensitivity analyses were performed by comparing CAR- and BAR-based models using identical covariate sets to assess the robustness of the associations. All statistical tests were two-tailed, and a *p*-value < 0.05 was considered statistically significant.

## 3. Results

During the study period, a total of 167 patients were initially assessed for eligibility. Sixteen patients were excluded based on the predefined exclusion criteria, resulting in a final cohort of 151 patients who underwent distal lower extremity amputation for diabetic foot infection. Among the excluded patients, the primary reason for exclusion was missing laboratory data required for the calculation of CAR and BAR. Follow-up duration was comparable between the groups. In the non-escalation group, follow-up ranged from 12 to 28 months (mean: 18.6 ± 4.5 months; median: 18 months), whereas in the level-escalation group it ranged from 11 to 29 months (mean: 17.7 ± 5.8 months; median: 15 months). There was no statistically significant difference between the groups in terms of follow-up duration (*p* = 0.85). Importantly, all observed major reamputation events occurred within the first six months of follow-up, indicating that the outcome represents an early postoperative phenomenon.

**Figure 1:**
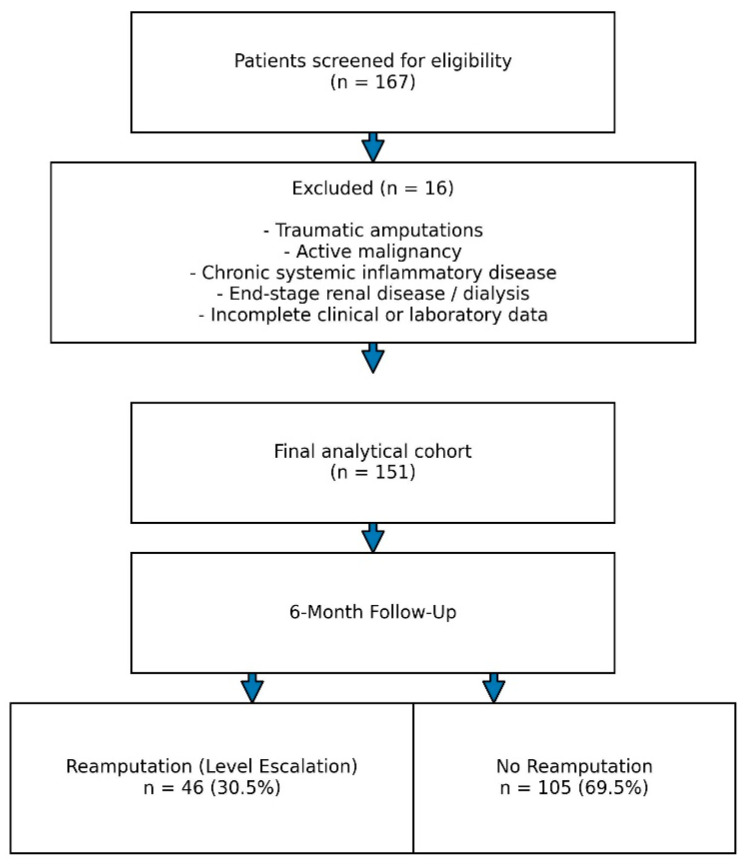
Flow diagram of patient selection.

During follow-up, 46 patients (30.5%) required major level escalation (below-knee amputation), whereas 105 patients (69.5%) did not require further surgical intervention. A flow diagram illustrating patient selection and study inclusion is presented in Figure 1.

Baseline demographic and clinical characteristics of the study population are presented in Table 1. There was no significant difference in age between patients who required major level escalation and those who did not (63.63 ± 11.84 vs. 64.11 ± 12.76 years, *p* = 0.623). Similarly, sex distribution (*p* = 0.131), amputation side (*p* = 0.544), presence of hypertension (*p* = 0.472), smoking status (*p* = 0.753), and peripheral arterial disease (*p* = 0.207) were comparable between the two groups, with no statistically significant differences observed.

In contrast, a significant difference was identified in terms of index amputation level (*p* < 0.001). Patients who subsequently required level escalation were more likely to have undergone a more proximal index amputation, whereas toe-level amputations were predominantly observed in the group without subsequent reamputation.

Preoperative laboratory parameters are summarized in Table 2. Patients who required major level escalation exhibited significantly higher HbA1c levels compared with those who did not (9.66 ± 1.94% vs. 8.74 ± 2.56%, *p* < 0.001). Similarly, markers of systemic inflammation were markedly elevated in the escalation group, including white blood cell count (12,824 ± 4685 vs. 10,403 ± 4327 /µL, *p* < 0.001), neutrophil count (10,090 ± 4414 vs. 7405 ± 4153 /µL, *p* < 0.001), C-reactive protein (112.48 ± 85.41 vs. 58.03 ± 82.98 mg/L, *p* < 0.001), and erythrocyte sedimentation rate (91 ± 28 vs. 70 ± 29 mm/h, *p* < 0.001).

Renal and metabolic stress markers were also higher in patients who underwent subsequent reamputation, with significantly elevated BUN levels (30.53 ± 18.12 vs. 24.24 ± 13.21 mg/dL, *p* = 0.041). In contrast, serum albumin levels were significantly lower in the level escalation group (29.63 ± 5.61 vs. 31.95 ± 4.14 g/L, *p* = 0.023).

When composite biomarkers were evaluated, both the BUN-to-albumin ratio and the CRP-to-albumin ratio were significantly higher in patients who subsequently required major level escalation. The mean BAR was 1.07 ± 0.63 in the escalation group compared with 0.78 ± 0.49 in the non-escalation group (*p* = 0.006). Similarly, the mean CAR was 4.07 ± 3.24 in patients requiring reamputation versus 1.96 ± 3.12 in those without level escalation (*p* < 0.001).

**Figure 2:**
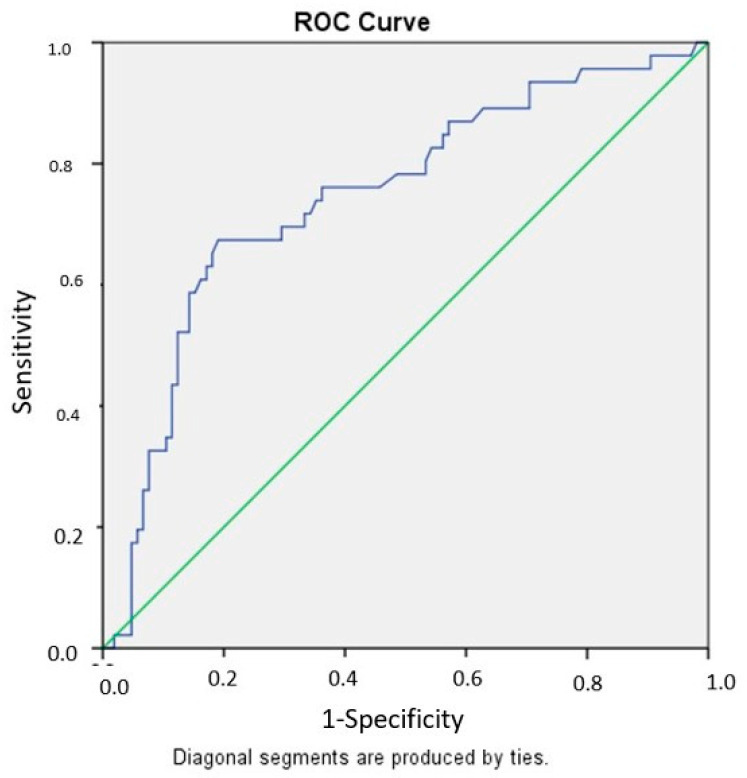
Receiver operating characteristic (ROC) curve of the CRP-to-albumin ratio (CAR) for predicting major level escalation. The area under the curve (AUC) was 0.745 (95% CI 0.653–0.827). The optimal cutoff value determined by the Youden index was 1.50, yielding a sensitivity of 76.1% and a specificity of 61.9%. These thresholds are presented as exploratory and are not intended for direct clinical decision-making.

**Figure 3:**
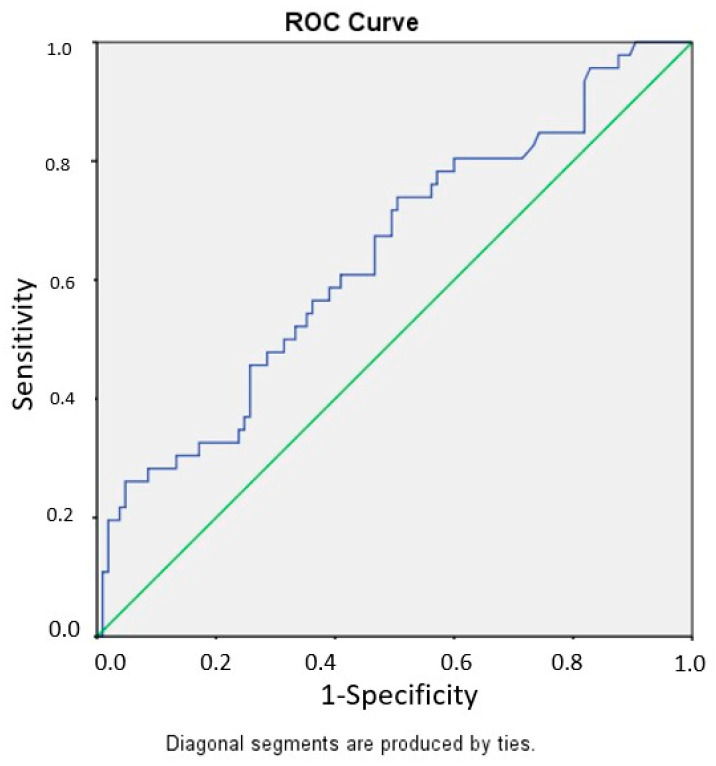
Receiver operating characteristic (ROC) curve of the BUN-to-albumin ratio (BAR) for predicting major level escalation. The area under the curve (AUC) was 0.640 (95% CI 0.536–0.738). The optimal cutoff value determined by the Youden index was 0.61, yielding a sensitivity of 73.9% and a specificity of 44.8%. These thresholds are presented as exploratory and are not intended for direct clinical decision-making.

Receiver operating characteristic (ROC) curve analysis demonstrated that CAR had acceptable discriminative performance for predicting level escalation (AUC = 0.745; 95% CI 0.653–0.827). In contrast, BAR showed modest discriminative ability (AUC = 0.640; 95% CI 0.536–0.738). Based on the Youden index, the optimal cutoff value for CAR was 1.50, yielding a sensitivity of 76.1% and a specificity of 61.9%. For BAR, the optimal cutoff value was 0.61, with a sensitivity of 73.9% and a specificity of 44.8%. These thresholds should be interpreted as exploratory and not intended for direct clinical decision-making. The corresponding ROC curves are presented in Figure 2 and Figure 3.

**Figure 4:**
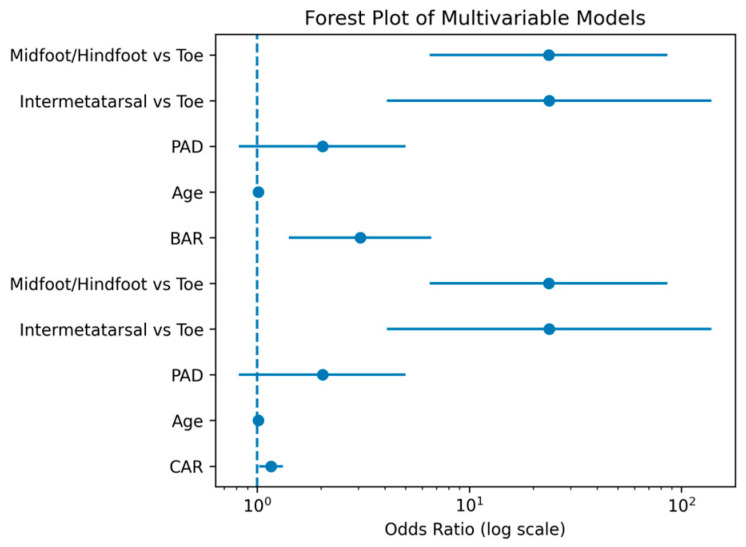
Forest plot of multivariable logistic regression analyses for predictors of major level escalation. Model A includes the CRP-to-albumin ratio (CAR), and Model B includes the BUN-to-albumin ratio (BAR). Odds ratios (OR) with 95% confidence intervals are shown. The vertical dashed line represents the reference value (OR = 1).

In multivariable logistic regression analysis, odds ratios and confidence intervals are illustrated in Figure 4. After adjustment for clinical covariates, CAR, BAR, and more proximal index amputation levels (intermetatarsal and midfoot/hindfoot) remained statistically significant predictors of major level escalation.

Two separate multivariable logistic regression models were constructed to evaluate the independent associations of CAR and BAR with major level escalation (Table 3). In Model A (CAR model), after adjustment for age, peripheral arterial disease (PAD), and index amputation level, the CRP-to-albumin ratio remained an independent predictor of level escalation (OR 1.21; 95% CI 1.08–1.37; *p* = 0.02).

In Model B (BAR model), after adjustment for the same clinical covariates, the BUN-to-albumin ratio was independently associated with major level escalation (OR 4.02; 95% CI 1.85–8.73; *p* = 0.005). In both models, index amputation level emerged as a strong independent determinant. Compared with toe-level amputations, intermetatarsal and midfoot/hindfoot amputations were associated with a significantly increased risk of subsequent level escalation. Age and peripheral arterial disease were not identified as independent predictors in either model.

**Figure 5:**
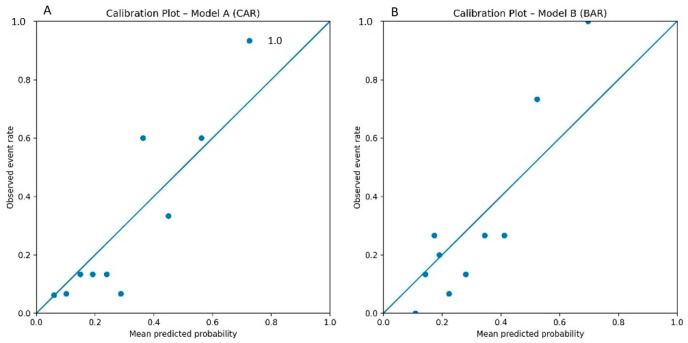
Calibration plots of the multivariable logistic regression models for post-amputation level escalation. (**A**) shows the calibration of Model A including CAR, and (**B**) shows the calibration of Model B including BAR. Observed event rates are plotted against mean predicted probabilities across deciles of predicted risk. The 45-degree reference line represents perfect calibration.

Odds ratios and corresponding 95% confidence intervals from the multivariable analyses are presented in forest plot format in Figure 4. Calibration plots for Model A (CAR) and Model B (BAR) are shown in Figure 5. Visual inspection of the calibration curves demonstrated good agreement between predicted probabilities and observed event rates for both models. The Hosmer–Lemeshow goodness-of-fit test was not significant for Model A (χ^2^ = 9.551, df = 8, *p* = 0.298) or Model B (χ^2^ = 14.434, df = 8, *p* = 0.071), indicating acceptable model calibration. Given the small number of events in these subgroups, these estimates should be interpreted with caution.

To further evaluate the robustness of the findings, sensitivity analyses were performed by constructing two additional multivariable logistic regression models including age, surgical amputation level, HbA1c, and white blood cell (WBC) count as covariates. In the model incorporating the BUN-to-albumin ratio (BAR), BAR remained independently associated with major reamputation (OR: 3.31, 95% CI: 1.57–6.99, *p* = 0.002). In contrast, in the model including the CRP-to-albumin ratio (CAR) with the same covariate set, CAR was not significantly associated with the outcome (OR: 1.13, 95% CI: 0.98–1.32, *p* = 0.100). These findings demonstrate that the association between BAR and major reamputation is robust and independent of clinically relevant confounders, whereas CAR does not retain independent predictive value under similar conditions.

Assessment of multicollinearity using the variance inflation factor (VIF) revealed values below 2 for all predictors, suggesting the absence of significant multicollinearity.

## 4. Discussion

In the present study, we found that postoperative major level escalation following distal amputation for diabetic foot is associated with composite biomarkers reflecting systemic inflammatory burden and nutritional/metabolic status. In the primary multivariable models, both CAR and BAR were independently associated with major level escalation after adjustment for age, PAD, and index amputation level. However, in sensitivity analyses incorporating additional covariates (HbA1c and WBC), only BAR retained independent significance, whereas the association of CAR was attenuated and no longer statistically significant. This differential robustness suggests that BAR may reflect a more stable and independent prognostic signal in this context. Findings suggest that major level escalation is not solely determined by local wound characteristics but is closely related to systemic physiological reserve. In addition, index amputation level emerged as a strong independent determinant, underscoring the critical role of initial surgical level in postoperative prognosis. Notably, in our cohort, all cases of major level escalation resulted in below-knee amputation, indicating that the observed associations relate to clinically meaningful major surgical outcomes rather than minor revisions. These findings should be interpreted as associations rather than evidence of predictive validation. The large odds ratios observed for more proximal amputation levels likely reflect the limited number of patients in these subgroups and the resulting wide confidence intervals. Therefore, these findings demonstrate an association rather than a precise predictive effect and should be interpreted with caution.

Inflammatory and nutritional biomarkers such as the CRP-to-albumin ratio (CAR) and the BUN-to-albumin ratio (BAR) have been widely investigated as prognostic indicators across various clinical settings, with demonstrated associations with mortality and complication risk [11,12]. However, their role in predicting the need for major reamputation following distal amputation in patients with diabetic foot has not been well established. In clinical practice, determining the appropriate level of amputation remains largely dependent on clinical judgment and subjective assessment, and there is currently no widely accepted and validated prognostic scoring system to guide this decision [13]. In this context, easily accessible biomarkers such as CAR and BAR may offer potential value as adjunctive tools for risk stratification.

CAR has been increasingly recognized as an integrated biomarker combining acute-phase inflammatory response and nutritional reserve [14]. Previous studies have suggested that CAR may predict the risk of major amputation in patients with diabetic foot infections [15]. However, most existing investigations have focused on the binary outcome of amputation versus limb salvage, without specifically addressing the need for more proximal reamputation following an initial distal procedure—an outcome with direct implications for functional prognosis. In this context, our study contributes to the literature by evaluating a more clinically nuanced endpoint. The acceptable discriminative performance of CAR observed in ROC analysis (AUC 0.745) suggests that it may have potential utility as a supportive parameter in preoperative risk assessment.

Although serum albumin alone is widely regarded as a marker of chronic inflammation and malnutrition, it may not adequately capture the acute inflammatory response accompanying severe infection [16]. The combination of CRP and albumin allows for simultaneous assessment of acute inflammatory activity and baseline nutritional reserve [17]. Considering that distal major level escalation in diabetic foot is likely influenced by persistent infection, microvascular dysfunction, and increased catabolic stress, CAR may reflect this multifactorial pathophysiological process more comprehensively than isolated laboratory parameters [18].

BAR, on the other hand, may be interpreted as an indirect indicator of systemic catabolic burden, renal perfusion alterations, and overall metabolic stress. Although its prognostic value has been demonstrated in critical illness and sepsis literature, data regarding its role in diabetic foot–related amputations remain limited. In our study, BAR was independently associated with major level escalation even after adjustment for clinical variables [8,19]. Despite demonstrating only modest discriminative performance in ROC analysis, its persistence as a significant predictor in multivariable modeling suggests that BAR may provide complementary prognostic information, particularly in reflecting systemic physiological reserve. In sensitivity analyses using identical covariate sets, BAR remained independently associated with major level escalation, whereas CAR did not retain statistical significance. This finding further supports the consistency of the observed association for BAR and suggests that its potential prognostic value is not solely driven by residual confounding.

Notably, the categorical analysis demonstrating a strong association between index amputation level and subsequent level escalation suggests that the initial surgical level may not fully reflect the true healing potential of the distal tissues. More proximal index amputations may represent a surrogate marker of extensive underlying infection or advanced vascular compromise. This finding supports the concept that surgical decision-making in diabetic foot should not rely solely on local tissue assessment but should also incorporate an evaluation of systemic inflammatory burden and overall physiological reserve.

Higher HbA1c levels observed in patients who developed level escalation reflect the adverse impact of chronic glycemic dysregulation on wound healing. Chronic hyperglycemia is well known to impair wound repair through mechanisms including microvascular dysfunction, dysregulated immune response, and reduced fibroblast proliferation [20]. Poor glycemic control has previously been associated with an increased risk of amputation in diabetic foot disease [21]. However, in our multivariable models, inflammatory and metabolic composite biomarkers remained independently associated with major level escalation, suggesting that acute inflammatory burden and systemic catabolic stress may exert a more immediate influence on distal amputation failure than chronic glycemic control alone. Guo et al. have emphasized the critical role of acute inflammatory response during the early phases of wound healing [5]. Within this framework, the pathophysiology of postoperative level escalation may involve not only chronic hyperglycemia but also acute systemic inflammation and impaired physiological reserve. These observations further imply that the biological mechanisms underlying initial amputation development and subsequent postoperative level escalation may differ. These findings should be interpreted within the broader pathophysiological framework of diabetes-related neurovascular injury, as current evidence suggests that early microstructural nerve alterations may precede overt clinical complications [22]. In this context, diabetic foot disease is increasingly recognized as a multifactorial condition involving a complex interplay between neuropathy, inflammation, and impaired wound healing. Although our study was not designed to directly evaluate these mechanisms, this framework may help to contextualize the observed associations [1].

From a clinical perspective, major level escalation following distal amputation has profound and lasting implications for functional capacity, prosthetic adaptation, and quality of life [4,23]. Therefore, ratios such as CAR and BAR—calculated from routinely obtained, inexpensive laboratory parameters— may provide supportive information in understanding risk patterns of postoperative major level escalation. Although ROC-derived thresholds were explored in this study, they should be interpreted with caution and are not intended for direct clinical application. However, these findings should be considered hypothesis-generating, and further studies with appropriate validation are required before these biomarkers can be incorporated into clinical decision-making. Future prospective, multicenter studies with standardized data collection are needed to validate these findings and to better define the role of these biomarkers in clinical decision-making.

### Limitations

This study has several limitations that should be acknowledged. First, its retrospective and single-center design limits the ability to fully exclude selection and measurement bias. Although amputation level decisions were made by a multidisciplinary team, inter-individual variability in clinical judgment may have influenced surgical decision-making and subsequent outcomes. Second, standardized vascular and infection severity assessments were not uniformly available. Objective perfusion parameters, such as the ankle–brachial index (ABI) or transcutaneous oxygen pressure (TcPO2), were not consistently recorded and therefore could not be incorporated into the regression models. Although Wagner classification was documented, it was not consistently integrated into the multivariable analysis. Given the central role of tissue perfusion and infection severity in wound healing and reamputation, residual confounding cannot be excluded. Third, although several clinically relevant variables—such as HbA1c, infection-related markers (e.g., WBC), and renal function parameters—were available, they were not included in the primary multivariable models. This approach was intentionally adopted to preserve model parsimony and minimize the risk of overfitting given the limited number of outcome events. However, this may have contributed to residual confounding and should be considered when interpreting the findings. Fourth, certain amputation subcategories included small numbers of patients, requiring the collapsing of categories for regression analyses. While this approach improved model stability and reduced sparse-data bias, it resulted in wide confidence intervals and should be considered when interpreting the magnitude of associations. Finally, although all patients were followed for at least six months and the majority of level escalation events occurred within this predefined period, time-to-event analysis was not performed. The retrospective design and lack of consistently recorded exact event timing limited the feasibility of survival analysis and the evaluation of temporal risk patterns.

## 5. Conclusions

In conclusion, preoperative BAR demonstrated consistent independent association with major level escalation following distal amputation in patients with diabetic foot, while CAR showed a significant association in the primary model that was not sustained in sensitivity analyses. As composite biomarkers reflecting systemic inflammatory burden and metabolic/nutritional reserve, these readily obtainable laboratory ratios may contribute to understanding associations with risk. However, these findings should be interpreted within the context of the study’s retrospective design and require validation in larger, prospective, and multicenter cohorts. These findings should be considered hypothesis-generating and require external validation before routine clinical application. Future investigations incorporating objective vascular parameters and external validation datasets are warranted to further clarify the clinical applicability of these biomarkers in surgical decision-making.

## Figures and Tables

**Table 1:** Baseline demographic and clinical characteristics of the study population.

Variable	No Reamputation (*n* = 105)	Reamputation (*n* = 46)	*p*-Value
Age (years)	63.6 ± 11.8	64.1 ± 12.8	0.623
Male sex	65 (61.9%)	35 (76.1%)	0.131
Right side	62 (59.0%)	24 (52.2%)	0.544
Hypertension	65 (61.9%)	32 (69.6%)	0.472
Smoking	41 (39.0%)	16 (34.8%)	0.753
Peripheral arterial disease	46 (43.8%)	26 (56.5%)	0.207
Index amputation level			<0.001
° Toe	99 (94.3%)	23 (50.0%)	
° Intermetatarsal	2 (1.9%)	8 (17.4%)	
° Lisfranc	0 (0.0%)	3 (6.5%)	
° Chopart	3 (2.9%)	5 (10.9%)	
° Syme	1 (1.0%)	7 (15.2%)	

**Table 2:** Preoperative laboratory parameters according to level escalation status.

Parameter (Unit)	No Reamputation (*n* = 105)	Reamputation (*n* = 46)	*p*-Value
HbA1c (%)	8.74 ± 2.56 (4.80–17.00)	9.66 ± 1.94 (5.30–16.50)	<0.001
WBC (/µL)	10,403 ± 4327	12,824 ± 4685	<0.001
Neutrophils (/µL)	7405 ± 4153	10,090 ± 4414	<0.001
Lymphocytes (/µL)	1975 ± 826	1632 ± 699	0.015
Platelets (/µL)	352,111 ± 145,412	387,413 ± 131,608	0.160
BUN (mg/dL)	24.24 ± 13.21	30.53 ± 18.12	0.041
Serum albumin (g/L)	31.95 ± 4.14	29.63 ± 5.61	0.023
BUN-to-albumin ratio	0.78 ± 0.49	1.07 ± 0.63	0.006
Creatinine (mg/dL)	1.28 ± 1.28 (0.41–7.50)	1.65 ± 1.68 (0.50–6.97)	0.158
CRP (mg/L)	58.03 ± 82.98 (0.20–438.00)	112.48 ± 85.41 (2.00–305.00)	<0.001
ESR (mm/h)	70 ± 29 (2–140)	91 ± 28 (22–140)	<0.001
CRP-to-albumin ratio	1.96 ± 3.12	4.07 ± 3.24	<0.001

**Table 3:** (**A**) Model A (CAR) Multivariable logistic regression analysis for predictors of level escalation. (**B**) Model B (BAR) Multivariable logistic regression analysis for predictors of level escalation.

(**A**)			
**Variable**	**OR**	**95% CI**	***p***
CRP/Albumin	1.21	1.08–1.37	0.024
Age	1.01	0.98–1.05	0.415
PAD	1.74	0.73–4.12	0.204
Intermetatarsal vs. Toe	16.27	3.02–87.60	<0.001
Midfoot/Hindfoot vs. Toe	18.27	5.30–63.03	<0.001
(**B**)			
**Variable**	**OR**	**95% CI**	***p***
BUN/Albumin	4.02	1.85–8.73	0.005
Age	1.008	0.97–1.04	0.675
PAD	2.08	0.86–5.02	0.102
Intermetatarsal vs. Toe	26.30	4.75–145.35	<0.001
Midfoot/Hindfoot vs. Toe	23.25	6.46–83.71	<0.001

## Data Availability

The datasets generated and/or analyzed during the current study are available from the corresponding author upon reasonable request.

## Abbreviations

The following abbreviations are used in this manuscript:

BAR	Blood urea nitrogen/albumin ratio
CAR	C-reactive protein/albumin ratio
BUN	Blood urea nitrogen
CRP	C-reactive protein
AUC	Area under the curve
PAD	Peripheral Arterial Disease
BKA	Below-Knee Amputation
VIF	Variance Inflation Factor
